# Effectiveness of nirsevimab against RSV-bronchiolitis in paediatric ambulatory care: a test-negative case–control study

**DOI:** 10.1016/j.lanepe.2024.101007

**Published:** 2024-07-23

**Authors:** Yannis Lassoued, Corinne Levy, Andreas Werner, Zein Assad, Stephane Bechet, Bruno Frandji, Christophe Batard, Aurélie Sellam, Fabienne Cahn-Sellem, Inès Fafi, Léa Lenglart, Camile Aupiais, Romain Basmaci, Robert Cohen, Naim Ouldali

**Affiliations:** a Department of General Paediatrics, Paediatric Infectious Disease and Internal Medicine, Robert Debré University Hospital, Assistance Publique-Hôpitaux de Paris, 75019, Paris, France; b Infection, Antimicrobials, Modelling, Evolution (IAME), INSERM UMR 1137, Paris Cité University, 75018, Paris, France; c Association Clinique et Thérapeutique Infantile du Val-de-Marne (ACTIV), Créteil, France; d Association Française de Pédiatrie Ambulatoire (AFPA), Paris, France; e Groupe de Pathologie Infectieuse Pédiatrique (GPIP), Créteil, France; f Université Paris Est, IMRB-GRC GEMINI, Créteil, France; g Clinical Research Center (CRC), Centre Hospitalier Intercommunal de Créteil, Créteil, France; h CompuGroup Medical, Nanterre, France; i General Paediatrics, Paediatric Emergency and Neonatal Intensive Care, Jean Verdier University Hospital, Assistance Publique-Hôpitaux de Paris, 93140, Bondy, France; j Paediatric Emergency Department, Robert Debré University Hospital, Assistance Publique-Hôpitaux de Paris, Paris Cité University, Paris, France; k ECEVE, Inserm UMR 1123, Paris Cité University, 75010, Paris, France; l General Paediatrics, Paediatric Emergency, Louis Mourier University Hospital, Assistance Publique-Hôpitaux de Paris, 92700, Colombes, France

**Keywords:** Bronchiolitis, RSV, Nirsevimab, Paediatric ambulatory care, Outpatient

## Abstract

**Background:**

Respiratory syncytial virus (RSV) is the leading cause of lower-respiratory-tract infection in children. Nirsevimab, a monoclonal antibody against RSV, was implemented in a few countries in September 2023. However, its post-license effectiveness in ambulatory care settings is unknown. We aimed to assess the effectiveness of nirsevimab against RSV-bronchiolitis in outpatients aged <12 months.

**Methods:**

We conducted a test-negative case–control study based on a national ambulatory surveillance system. We included all infants aged <12 months who had bronchiolitis and results of an RSV rapid antigen test performed, visiting a network of 107 ambulatory paediatricians from September 15, 2023, to February 1, 2024. Case patients were infants with bronchiolitis and a rapid antigen test positive for RSV. Control patients were infants with bronchiolitis and a rapid antigen test negative for RSV. Effectiveness was assessed by a logistic regression model adjusted for potential confounders. A range of sensitivity analyses were conducted to assess the robustness of the findings.

**Findings:**

We included 883 outpatients who had bronchiolitis and results of an RSV rapid antigen test (453 were case patients, and 430 were control patients). Overall, 62/453 (13.7%) case patients and 177/430 (41.2%) control patients had been previously immunised for nirsevimab. The adjusted effectiveness of nirsevimab against RSV-bronchiolitis was 79.7% (95% CI 67.7–87.3). Sensitivity analyses gave similar results.

**Interpretation:**

This post-license study indicates that nirsevimab was effective in preventing RSV-bronchiolitis in ambulatory care settings.

**Funding:**

The study was supported by Association Clinique et Thérapeutique Infantile du Val de Marne (ACTIV), French Pediatrician Ambulatory Association (AFPA) and unrestricted grants from GSK, 10.13039/100030732MSD, 10.13039/100004319Pfizer and 10.13039/100004339Sanofi.

**Research in context**

**Evidence before this study**

We searched PubMed, supplemented with internet searches (Google), for studies evaluating the real-world effectiveness of nirsevimab up to March 10, 2024. We used the terms “effectiveness” or “effect” or “impact” or “efficacy” or “association” AND “nirsevimab” AND “RSV bronchiolitis”. We found several post-license studies evaluating the real-world effectiveness of nirsevimab against respiratory syncytial virus (RSV)-bronchiolitis, in the United States, Spain and France. These studies found an effectiveness ranging from 70% to 90%. However, these studies were limited to hospitalised patients. The effectiveness of nirsevimab in ambulatory care settings remains to be assessed.

**Added value of this study**

We conducted a test-negative case–control study based on a national ambulatory surveillance system to assess the effectiveness of nirsevimab against RSV-bronchiolitis in paediatric ambulatory care among children aged <12 months. We performed the study over the 2023–2024 RSV season. This study highlights a strong effectiveness of nirsevimab in preventing RSV-bronchiolitis in ambulatory care settings, close to that found in inpatient settings. We suggest that the benefit of nirsevimab also extends to ambulatory forms of RSV bronchiolitis, which are by far the most frequent forms of RSV infections.

**Implications of all the available evidence**

Most countries are currently considering adding nirsevimab to national immunisation programs. In this context, this real-world effectiveness study may guide policymakers when assessing the potential benefit of nirsevimab in preventing RSV-bronchiolitis.

## Introduction

Bronchiolitis is the most common lower-respiratory-tract infection in children.^1^ Respiratory syncytial virus (RSV) is the main pathogen involved in bronchiolitis, responsible for approximately 33 million cases, with 3.2 million hospitalizations and up to 200,000 deaths each year globally.^2^ Overall, 20% of infants <2 years old are affected by RSV-bronchiolitis, and 80%–90% of cases are managed in ambulatory care settings.^3^, ^4^, ^5^, ^6^

Palivizumab has been the only preventive treatment for RSV, indicated only for premature or high-risk infants.^7^ Recently, nirsevimab, an extended half-life recombinant monoclonal antibody binding the RSV prefusion protein and neutralising viral entry into host cells has been developed.^8^ Results from randomized, double-blind, placebo-controlled phase 3 trials showed its efficacy against RSV-associated lower-respiratory-tract infections.^9^, ^10^, ^11^ Thereafter, nirsevimab was approved by the US Food and Drug Administration and the European Medicines Agency in 2023.^12^^,^^13^ Nirsevimab was implemented in September 2023 in a few countries.^14^^,^^15^ However, the effectiveness of nirsevimab against RSV-bronchiolitis in ambulatory care settings is unknown.

The aim of our study was to assess the real-world effectiveness of nirsevimab against RSV-bronchiolitis in paediatric ambulatory care in infants aged <12 months over an epidemic season.

## Methods

### Study design

We conducted a national-based test-negative case–control study from September 15, 2023, to February 1, 2024, of outpatients aged <12 months with a diagnosis of bronchiolitis. The test-negative design has been increasingly used to estimate the effectiveness of immunisation programs.^16^, ^17^, ^18^ As compared with classical case–control studies, it reduces bias due to differences in healthcare-seeking behaviour or access to testing.^19^^,^^20^

### Settings and data collection

This study was based on the national Paediatric and Ambulatory Research in Infectious diseases (PARI) network. PARI is a French surveillance system dedicated to ambulatory-care paediatric infectious diseases, involving 107 ambulatory paediatricians across the French territory who are specifically trained in infectious diseases, with harmonized practices in accordance with international infectious disease guidelines.^21^^,^^22^ All infectious diseases diagnosed by any paediatrician are automatically collected and included in this surveillance system. Diagnoses are recorded according to the International Classification of Diseases and Related Health Problems, Tenth Revision (ICD-10). Clinical and demographic data were collected for each patient, along with the results of RSV rapid antigen tests^23^^,^^24^ and immunisation status. All collected data were anonymous. Details of the PARI network are available in the Supplementary Appendix. The study was approved by the French National Commission on Informatics and Liberty (no. 1921226) and by an ethics committee (CHI Creteil Hospital, France) and was registered at ClinicalTrials.gov (NCT04471493).

### Nirsevimab immunisation campaign in France

France was one of the first countries to implement a national immunisation campaign for nirsevimab in winter 2023–2024. French health authorities recommended a single dose of nirsevimab administered to all infants born after February 6, 2023.^14^ During the 2023–2024 epidemic, more than 230,000 doses were administered in France,^25^ which had 678,000 births each year.^26^ The national immunisation campaign began on September 15, 2023, before the start of the RSV epidemic (Figure S1, Supplementary Appendix).^27^ Because of a similar efficacy and safety profile as palivizumab but a simpler administration regimen, immunisation with nirsevimab was also recommended for all infants aged <12 months who were eligible for palivizumab.^28^ Due to national shortage, priority was given to allocate doses to newborns and children with risk factors for severe bronchiolitis.

### Inclusion and exclusion criteria

We included all outpatients aged <12 months who had a diagnosis of bronchiolitis (ICD-10 code: J21, J21.0, J21.8, J21.9) according to the international definition,^29^^,^^30^ who were visiting one of the participating ambulatory paediatricians between September 15, 2023 and February 1, 2024 and for whom an RSV rapid antigen test was performed (COVID-VIRO ALL-IN TRIPLEX, AAZ-LMB, Boulogne-Billancourt, France). Case patients were defined as infants with bronchiolitis and a rapid antigen test positive for RSV. Control patients were defined as infants with bronchiolitis and a rapid antigen test negative for RSV. We excluded infants included in the HARMONIE study, which took place in winter 2022–2023. We also excluded infants with previous immunisation with palivizumab or maternal vaccination against RSV. The maternal immunisation program against RSV has not been implemented in France.

### Outcomes

The primary outcome was RSV-positive bronchiolitis in infants aged <12 months. The exposure was nirsevimab immunisation status among case and control patients (occurring at any time before the diagnosis). We performed subgroup analyses by age group (<3 months, 3–6 months, >6 months) and premature birth (defined as gestational age <37 weeks).

### Sample size calculation

On the basis of an expected nirsevimab coverage of 25% for the general population of infants aged <12 months in France, the study sample size was calculated to detect a 50% reduction in the odds of nirsevimab immunisation among case patients versus control patients. Assuming a two-sided α of 0.05 and a power of 0.90, we needed 268 patients in each group (i.e., 536 patients in total).

### Statistical analysis

The effectiveness of nirsevimab against RSV-bronchiolitis was estimated by multivariate logistic regression adjusted for potential confounders comparing the likelihood of nirsevimab immunisation among case and control patients. The multivariate regression model was adjusted for age, sex, birth term, birth weight, previous bronchiolitis, number of children per household, month of diagnosis, childcare settings, and region. We performed multiple imputation by a chained equation generating 20 replicates to include missing data for the main and subgroup analyses. The effectiveness of nirsevimab was estimated with the following equation: 100% x (1- adjusted odds ratio).

We performed the following sensitivity analyses to assess the robustness of the study findings. First, we conducted an analysis adjusted for paediatrician investigators using a mixed effect logistic regression model, with investigator treated as a random effect to account for a potential centre effect. Second, we performed a multivariate model excluding patients with previous bronchiolitis to account for potential misdiagnosis between bronchiolitis and recurrent wheezing or another respiratory disease. Third, we conducted a complete case analysis to handle missing data. Fourth, to account for a potential remaining indication bias of nirsevimab administration, we conducted a propensity score analysis, using the inverse probability of weighting method. Each observation was weighted by the inverse of the propensity to be immunised (if the patient received nirsevimab) or non-immunised (if the patient did not receive nirsevimab). All covariates included in the main analysis were used to build the propensity score. Fifth, we performed an additional analysis by converting continuous variables into categorial variables. Sixth, a matched case–control analysis based on the diagnosis date was conducted, with a 1:1 ratio. Seventh, the multivariate analysis was adjusted for the week of diagnosis (instead of the month) to better account for the rapidly increasing national coverage of nirsevimab over the study period. Eight, we conducted a multivariate analysis adjusted for the month of birth to account for the higher likelihood of a severe RSV infection for infants born closer to the onset of the RSV season. Ninth, we performed a multivariate analysis excluding patients with bronchiolitis diagnosed within 7 days of nirsevimab injection to account for the length of incubation of RSV. All sensitivity analyses are detailed in the Supplementary Appendix (Table S1 and Figure S2).

The tests were two-sided, with level of significance p < 0.05. Analyses were performed with R v4.3.2.

### Role of the funding source

The funders of the study had no role in the study design, data collection, data analysis, data interpretation, or writing of the report.

## Results

**Fig. 1:**
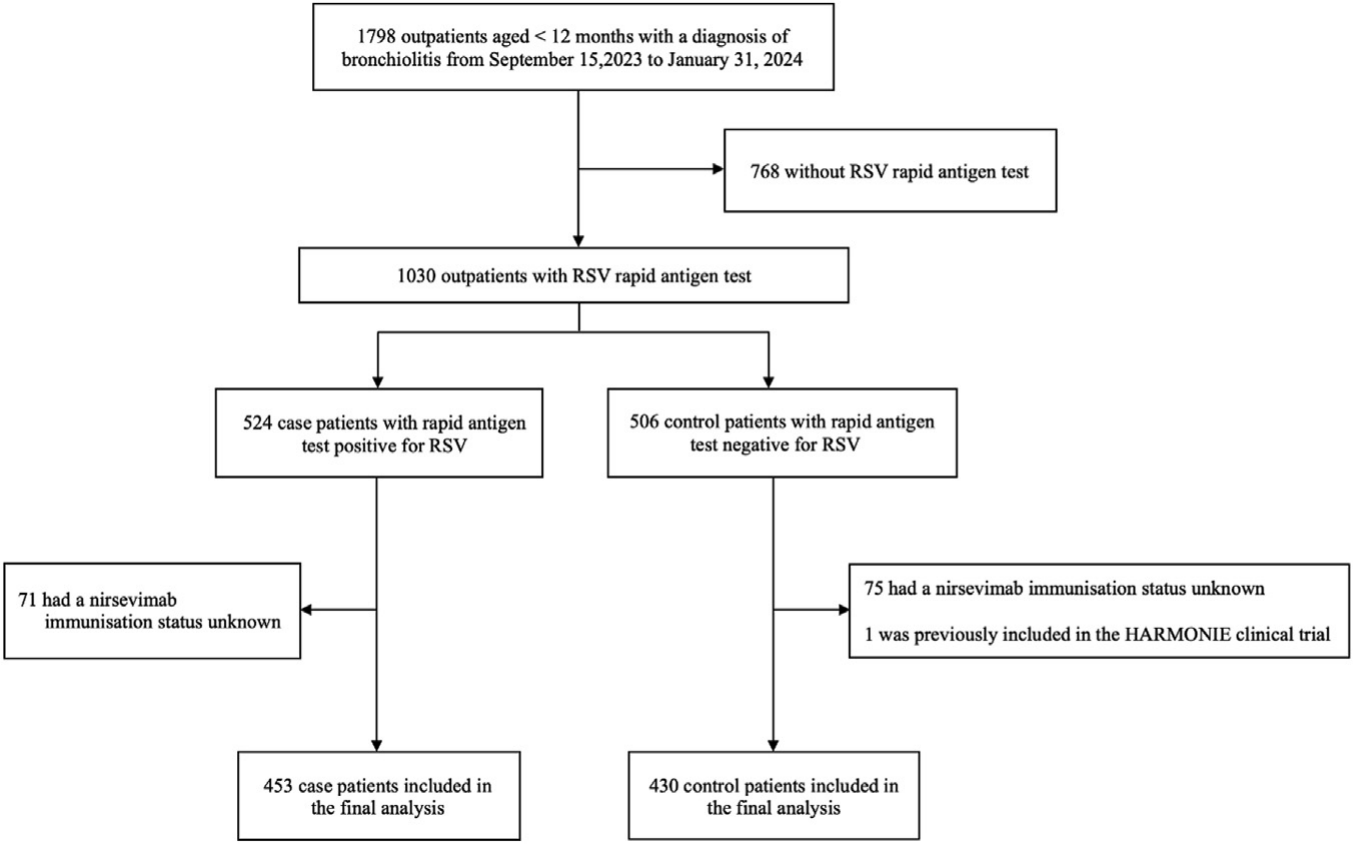
**Flow chart of participants in the study**. Abbreviation: RSV, respiratory syncytial virus.

Between September 15, 2023, and February 1, 2024, 1798 outpatients aged <12 months had a diagnosis of bronchiolitis within the PARI network (Figure S3, Supplementary Appendix). Among them, 1030 infants had an RSV rapid antigen test, and 883 patients were included in the final analysis: 453 case patients and 430 control patients (Fig. 1). Characteristics of included and excluded patients were similar, especially infants with and without RSV rapid antigen test results (Table S2, Supplementary Appendix). The proportion of patients immunised with nirsevimab over the study period are available in Figures S4 and S5, Supplementary Appendix.

Clinical characteristics are detailed in Table 1. The median age for case patients and control patients was 7.2 months (IQR 4.6–9.8) and 6.3 months (IQR 4.7–8.2). The proportion of preterm births was similar between case patients and control patients (32/358, 8.9% and 3/352, 9.4%).

**Table 1:** Characteristics of cases and controls (N = 883).

Characteristic	Cases (N = 453)	Controls (N = 430)
**Age at diagnosis, median (IQR), months**	7.2 (4.6–9.8)	6.3 (4.7–8.2)
**Age group (%)**		
<3 months	53/453 (11.7)	35/430 (8.2)
3 to 6 months	126/453 (27.8)	164/430 (38.1)
>6 months	274/453 (60.5)	231/430 (53.7)
**Sex (%)**		
Male	269/453 (59.4)	279/430 (64.9)
Female	184/453 (40.6)	151/430 (35.1)
**Birth term, median (IQR), weeks' gestational age**	39 (38–40) (NA = 95)	39 (38–40) (NA = 78)
**Preterm birth (%)**^a^	32/358 (8.9)	33/352 (9.4)
Late preterm 34–36 weeks	25/32 (78.1)	21/33 (63.6)
Moderate preterm 32–33 weeks	4/32 (12.5)	5/33 (15.1)
Very preterm 28–31 weeks	2/32 (6.2)	7/33 (21.2)
Extremely preterm < 28 weeks	1/32 (3.1)	7/33 (21.2)
**Birth weight, median (IQR), kg**	3.32 (3.03–3.66) (NA = 302)	3.22 (2.95–3.61) (NA = 262)
**History of bronchiolitis (%)**		
None	410/453 (90.5)	367/430 (85.3)
1 episode	37/453 (8.2)	56/430^13^
≥2 episodes	6/453 (1.3)	7/430 (1.6)
**Month of diagnosis (%)**		
September, 2023	14/453 (3.1)	31/430 (7.2)
October, 2023	65/453 (14.3)	107/430 (24.9)
November, 2023	227/453 (50.1)	113/430 (26.3)
December, 2023	132/453 (29.1)	111/430 (25.8)
January, 2024	15/453 (3.3)	68/430 (15.8)
**Childcare setting (%)**		
Home	141/322 (43.8)	143/309 (46.3)
Childminder	62/322 (19.2)	61/309 (19.7)
Day care centre/School	119/322 (37)	105/309 (34)
**Children per household (%)**		
1	111/307 (36.1)	100/288 (34.7)
2	136/307 (44.3)	140/288 (48.6)
≥3	60/307 (19.5)	48/288 (16.6)

Case patients were outpatients aged <12 months with bronchiolitis and a rapid antigen test positive for RSV. Control patients were outpatients aged <12 months with bronchiolitis and a rapid antigen test negative for RSV.

Abbreviations: NA, not available; IQR, interquartile range; RSV, respiratory syncytial virus.

a Preterm birth was defined as birth before 37 weeks of gestation.

### Primary outcome

**Fig. 2:**
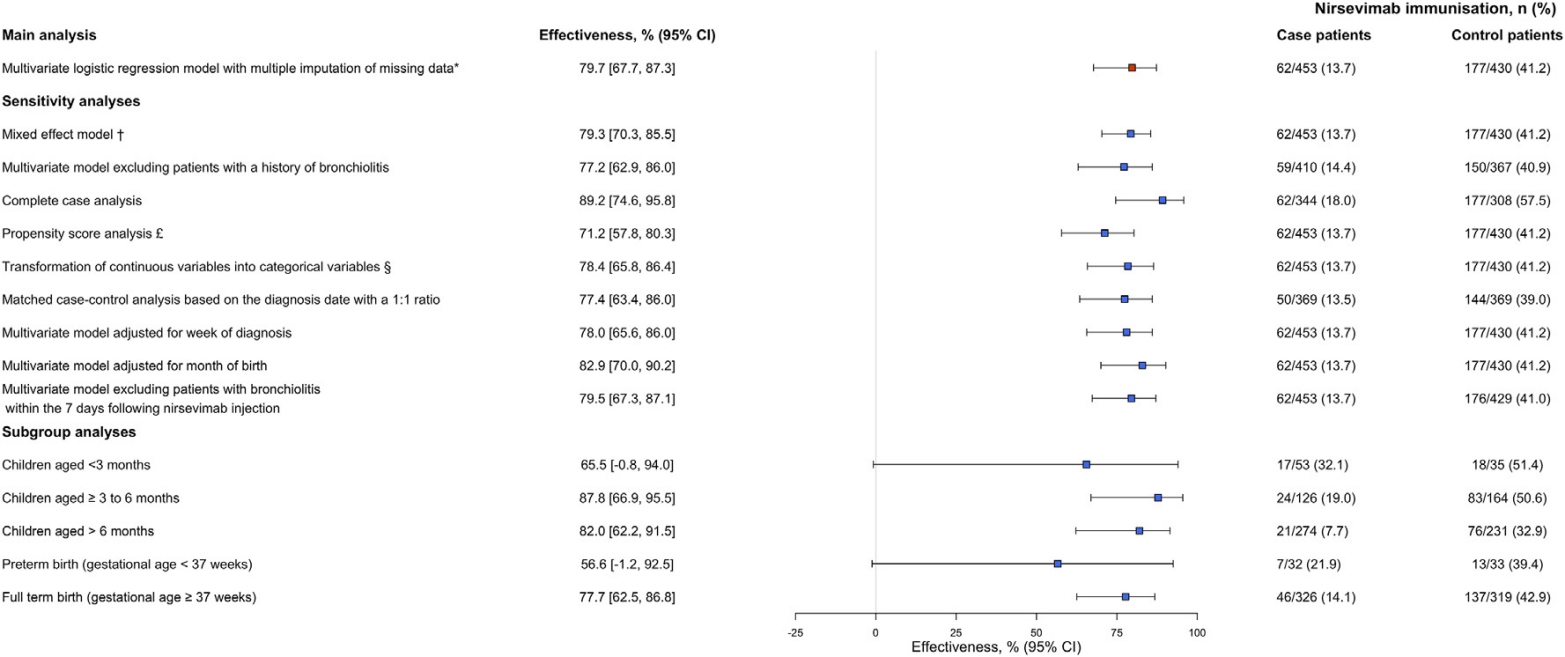
**Effectiveness of nirsevimab against RSV-bronchiolitis in paediatric ambulatory care**. Case patients were outpatients aged <12 months with bronchiolitis who had a rapid antigen test positive for RSV. Control patients were outpatients aged <12 months with bronchiolitis who had a rapid antigen test negative for RSV. Effectiveness was calculated as 100% x (1 − adjusted odds ratio). All sensibility analyses involved using multiple imputation, except for the complete case analysis, and is detailed in Table S1, Supplementary Appendix. ∗ Multivariate logistic regression model adjusted for age, sex, birth term, birth weight, history of bronchiolitis, number of children per household, region, childcare settings, and month of diagnosis. † Mixed effect model with multiple imputation. Investigator was treated as a random effect. £ All covariates of the main analysis were included in the propensity score (Figure S2, Supplementary Appendix). § Birth weight was classified into quartiles and birth term was dichotomised as birth < or ≥37 weeks' gestational age. Abbreviations: RSV, respiratory syncytial virus; CI, confidence interval.

Overall, 62/453 (13.7%) case patients were immunised with nirsevimab versus 177/430 (41.2%) control patients. The adjusted effectiveness of nirsevimab against RSV-bronchiolitis was 79.7% (95% confidence interval [CI], 67.7–87.3). All sensitivity analyses gave similar results (Fig. 2).

### Subgroup analysis

The effectiveness of nirsevimab for infants aged <3 months, 3–6 months and >6 months was 65.5% (95% CI, −0.8 to 94.0), 87.8% (95% CI, 66.9–95.5), and 82.0% (95% CI, 62.2–91.5), respectively (Fig. 2). Its effectiveness for infants born preterm was 56.6% (95% CI, −1.2 to 92.5) versus 77.7% (95% CI, 62.5–86.8) for full term-born infants.

## Discussion

This real-world study assessed the effectiveness of nirsevimab against RSV-bronchiolitis in infants aged <12 months in ambulatory care settings, highlighting an overall effectiveness of 79.7% (95% CI, 67.7–87.3).

Few post-license studies have been published on the effectiveness of nirsevimab, all of them focusing on hospitalised patients. In France, a case-control study of 1035 hospitalized infants <12 months found a 83.0% (95% CI 73.4–89.2) effectiveness of nirsevimab against RSV-bronchiolitis.^15^ In the United States, a test-negative case–control study of 699 infants aged <8 months who were hospitalised for acute respiratory infection between October 2023 and February 2024 showed 90% effectiveness of nirsevimab against RSV-associated hospitalisations.^31^ In Spain, a fisrt study of 166 patients with a similar design estimated the nirsevimab effectiveness at 70%–84%, with disparities between centres, notably because of different case definitions.^32^ Another Spanish study, NIRSE-GAL study, performed in hospital settings showed an effectiveness of 82.0% (95% CI 65.6–90.2) against RSV bronchiolitis hospitalisations, 86.9% (95% CI 69.1–94.2) against severe RSV bronchiolitis requiring oxygen support and 69.2% (55.9–78.0) against all-cause of low respiratory tract infections hospitalisations.^33^ All these studies involved patients hospitalised for RSV severe respiratory diseases. The present study, focusing on outpatients only, found a similar effectiveness, which suggests that the benefit of nirsevimab also extends to ambulatory forms of RSV-bronchiolitis, which are by far the most frequent forms of RSV infections.

Because of the very recent implementation of nirsevimab, our study evaluated the short-term effectiveness of this immunisation program. The long-term benefit needs to be further explored. Nevertheless, our study was able to cover the entire 2023–2024 RSV season and stopped when the RSV circulation was very low.^27^ Thus, it allowed for estimating the benefit of an immunisation program aiming to cover an entire RSV season.

This study has several limitations. First, the observational design did not allow for causative conclusions. Nevertheless, the use of a test-negative design allowed for reducing the risk of selection biases of standard case–control studies.^17^^,^^19^^,^^20^ Because case patients and control patients consulted for similar symptoms, the risk of bias related to health-seeking behaviours may be limited.^19^^,^^34^

Second, some outpatients screened for the study were excluded because they were not tested for RSV, which could have introduced selection bias. However, patient characteristics were similar between included and excluded patients, especially patients tested and not tested for RSV. Notably, the proportion of nirsevimab immunisation among infants tested and not tested for RSV was similar.

Third, because RSV detection was based on a rapid antigen test, we cannot exclude false-negative or false-positive cases. However previous studies showed that the sensitivity and specificity of the RSV rapid antigen test is 79.1% and 100%, respectively, thereby limiting the risk of misclassification.^23^^,^^24^ Furthermore, rapid antigen tests have the benefit of providing results during the visit.

Fourth, data regarding congenital heart defects were not available. However, the frequency of this condition in the general paediatric population is close to 1%.^35^

Fifth, due to the study design, clinical evolution of patients was not assessed, especially the rate of patients requiring hospitalisations.

### Conclusion

This post-license study found that nirsevimab was effective against RSV-bronchiolitis in infants aged <12 months during their first RSV season in ambulatory paediatric care. These findings support the large-scale implementation of nirsevimab in the general paediatric population. Further medico-economic studies are required to assess the cost benefit of nirsevimab immunoprophylaxis.

## Contributors

YL, RC and NO take responsibility for the content of the manuscript, including the integrity of the data and the accuracy of the data analysis. NO, RC, CL, and YL conceptualised the study. YL led the literature review. YL and SB led the data analysis and visualisation. YL, RC and NO led the data interpretation. YL and NO drafted the manuscript. CL, RC, AW, ZA, BF, AS, FCS, IF, LL, CA and RB commented on the draft report. All authors contributed to critical revision of the manuscript for important intellectual content. All authors had full access to all the aggregated data in the study. All authors read and approved the final draft of the manuscript and had final responsibility for the decision to submit for publication.

## Data sharing statement

Anonymized data are available on reasonable request to the principal investigator (NO).

## Declaration of interests

YL has no conflicts of interest to disclose. RB declares receiving fees from Sanofi and MSD for medical conferences or scientific meetings. CL declares receiving travel grants from MSD, Pfizer and fees from MSD and Pfizer for scientific meetings and expert board participation. AW declares receiving fees from Sanofi, GSK and MSD for medical conferences or scientific meetings. FCS declares receiving fees from Sanofi for expert board participation. CB declares receiving fees from Sanofi, GSK and MSD for medical conferences or scientific meetings. RC reports personal fees and non-financial support from Pfizer and personal fees from GSK, Merck, Pfizer, Sanofi, Viatris outside the submitted work. NO declares receiving travel grants from MSD, Pfizer, Sanofi, and GSK.

## References

[1] Dalziel S.R., Haskell L., O'Brien S. (2022). Bronchiolitis. Lancet.

[2] Shi T., McAllister D.A., O'Brien K.L. (2017). Global, regional, and national disease burden estimates of acute lower respiratory infections due to respiratory syncytial virus in young children in 2015: a systematic review and modelling study. Lancet.

[3] Hall C.B., Weinberg G.A., Iwane M.K. (2009). The burden of respiratory syncytial virus infection in young children. N Engl J Med.

[4] Stockman L.J., Curns A.T., Anderson L.J., Fischer-Langley G. (2012). Respiratory syncytial virus-associated hospitalizations among infants and young children in the United States, 1997-2006. Pediatr Infect Dis J.

[5] Hall C.B., Weinberg G.A., Blumkin A.K. (2013). Respiratory syncytial virus-associated hospitalizations among children less than 24 months of age. Pediatrics.

[6] Meissner H.C. (2016). Viral bronchiolitis in children. N Engl J Med.

[7] Garegnani L., Styrmisdóttir L., Roson Rodriguez P., Escobar Liquitay C.M., Esteban I., Franco J.V. (2021). Palivizumab for preventing severe respiratory syncytial virus (RSV) infection in children. Cochrane Database Syst Rev.

[8] Zhu Q., McLellan J.S., Kallewaard N.L. (2017). A highly potent extended half-life antibody as a potential RSV vaccine surrogate for all infants. Sci Transl Med.

[9] Domachowske J., Madhi S.A., Simões E.A.F. (2022). Safety of nirsevimab for RSV in infants with heart or lung disease or prematurity. N Engl J Med.

[10] Hammitt L.L., Dagan R., Yuan Y. (2022). Nirsevimab for prevention of RSV in healthy late-preterm and term infants. N Engl J Med.

[11] Simões E.A.F., Madhi S.A., Muller W.J. (2023). Efficacy of nirsevimab against respiratory syncytial virus lower respiratory tract infections in preterm and term infants, and pharmacokinetic extrapolation to infants with congenital heart disease and chronic lung disease: a pooled analysis of randomised controlled trials. Lancet Child Adolesc Health.

[12] Commissioner O of the. FDA. FDA (2023). FDA Approves New Drug to prevent RSV in Babies and Toddler.

[13] New medicine to protect babies and infants from respiratory syncytial virus (RSV) infection | European Medicines Agency. https://www.ema.europa.eu/en/news/new-medicine-protect-babies-and-infants-respiratory-syncytial-virus-rsv-infection.

[14] (2023). Réponses Rapides : Nirsévimab (Beyfortus®) dans la prévention des bronchiolites à virus respiratoire syncytial (VRS) chez les nouveau-nés et les nourrissons.

[15] Assad Z., Romain A.S., Aupiais C. (2024). Nirsevimab and hospitalization for RSV bronchiolitis. N Engl J Med.

[16] Feng S., Chiu S.S., Chan E.L.Y. (2018). Effectiveness of influenza vaccination on influenza-associated hospitalisations over time among children in Hong Kong: a test-negative case-control study. Lancet Respir Med.

[17] Thompson M.G., Stenehjem E., Grannis S. (2021). Effectiveness of Covid-19 vaccines in ambulatory and inpatient care settings. N Engl J Med.

[18] Carazo S., Skowronski D.M., Brisson M. (2023). Protection against omicron (B.1.1.529) BA.2 reinfection conferred by primary omicron BA.1 or pre-omicron SARS-CoV-2 infection among health-care workers with and without mRNA vaccination: a test-negative case-control study. Lancet Infect Dis.

[19] Chua H., Feng S., Lewnard J.A. (2020). The use of test-negative controls to monitor vaccine effectiveness: a systematic review of methodology. Epidemiology.

[20] Lewnard J.A., Tedijanto C., Cowling B.J., Lipsitch M. (2018). Measurement of vaccine direct effects under the test-negative design. Am J Epidemiol.

[21] Cohen R., Béchet S., Gelbert N. (2021). New approach to the surveillance of pediatric infectious diseases from ambulatory pediatricians in the digital era. Pediatr Infect Dis J.

[22] Cohen P.R., Rybak A., Werner A. (2022). Trends in pediatric ambulatory community acquired infections before and during COVID-19 pandemic: a prospective multicentric surveillance study in France. Lancet Reg Health Eur.

[23] Ferrani S., Prazuck T., Béchet S., Lesne F., Cohen R., Levy C. (2023). Diagnostic accuracy of a rapid antigen triple test (SARS-CoV-2, respiratory syncytial virus, and influenza) using anterior nasal swabs versus multiplex RT-PCR in children in an emergency department. Infect Dis Now.

[24] Cohen R., Haas H., Romain O. (2024). Use of rapid antigen triple test nasal swabs (COVID-VIRO ALL-IN TRIPLEX: severe acute respiratory syndrome coronavirus 2, respiratory syncytial virus, and influenza) in children with respiratory symptoms: a real-life prospective study. Open Forum Infect Dis.

[25] Borel C. (2023). https://www.omedit-idf.fr/immunisation-contre-le-vrs-beyfortus/.

[26] Naissances – fécondité–France–TABLEAU DE BORD DE L’économie française. https://www.insee.fr/fr/outil-interactif/5367857/tableau/20_DEM/22_NAI.

[27] Bronchiolite santé publique France. https://www.santepubliquefrance.fr/maladies-et-traumatismes/maladies-et-infections-respiratoires/bronchiolite.

[28] AFPA Association Française de Pédiatrie Ambulatoire Avis de la SFN et du GPIP sur la prévention des infections respiratoires basses à VRS dans la population néonatale, y compris la population des nouveau-nés prématurés–AFPA. https://afpa.org/recommandation/avis-de-la-societe-francaise-de-neonatologie-et-du-groupe-de-pathologie-infectieuse-pediatrique-sur-la-prevention-des-infections-respiratoires-basses-a-vrs-dans-la-population-neonatale-y-compris-la-p/.

[29] American Academy of Pediatrics Subcommittee on Diagnosis and Management of Bronchiolitis (2006). Diagnosis and management of bronchiolitis. Pediatrics.

[30] Haute Autorité de Santé Prise en charge du 1er épisode de bronchiolite aiguë chez le nourrisson de moins de 12 mois. https://www.has-sante.fr/jcms/p_3118113/fr/prise-en-charge-du-1er-episode-de-bronchiolite-aigue-chez-le-nourrisson-de-moins-de-12-mois.

[31] Moline H.L., Tannis A., Toepfer A.P. (2024). Early estimate of nirsevimab effectiveness for prevention of respiratory syncytial virus–associated hospitalization among infants entering their first respiratory syncytial virus season — new vaccine surveillance network, October 2023–February 2024. MMWR Morb Mortal Wkly Rep.

[32] López-Lacort M., Muñoz-Quiles C., Mira-Iglesias A. (2024). Early estimates of nirsevimab immunoprophylaxis effectiveness against hospital admission for respiratory syncytial virus lower respiratory tract infections in infants, Spain, October 2023 to January 2024. Euro Surveill.

[33] Ares-Gómez S., Mallah N., Santiago-Pérez M.I. (2024). Effectiveness and impact of universal prophylaxis with nirsevimab in infants against hospitalisation for respiratory syncytial virus in Galicia, Spain: initial results of a population-based longitudinal study. Lancet Infect Dis.

[34] De Serres G., Skowronski D.M., Wu X.W., Ambrose C.S. (2013). The test-negative design: validity, accuracy and precision of vaccine efficacy estimates compared to the gold standard of randomised placebo-controlled clinical trials. Euro Surveill.

[35] CDC. Centers for Disease Control and Prevention (2023). https://www.cdc.gov/ncbddd/heartdefects/data.html.

