# Supplementary material for: Effectiveness of nirsevimab against RSV-bronchiolitis in paediatric ambulatory care: a test-negative case–control study
Source: Lancet Reg Health Eur. 2024 Jul 23;44:101007. doi: 10.1016/j.lanepe.2024.101007 (PMC11321316; doi:10.1016/j.lanepe.2024.101007)

**Supplementary Materials**

**Effectiveness of nirsevimab against RSV-bronchiolitis in paediatric ambulatory care: a test-negative case–control study**

**Figure S1. Respiratory syncytial virus (RSV) positivity rate for nasopharyngeal or salivary swabs collected in France during 2018-2019 to 2023-2024.**

**Supplementary information. The Pediatric and Ambulatory Research in Infectious diseases (PARI) network.**

**Table S1. Sensitivity analyses for the effectiveness of nirsevimab against respiratory syncytial virus (RSV)-bronchiolitis.**

**Figure S2. Covariate balance for the propensity score analysis.**

**Table S2. Characteristics of included and excluded patients.**

**Figure S3. Monthly number of case and control patients included over the study period.**

**Figure S4. Proportion of patients immunised with nirsevimab over the study period.**

**Figure S5. Proportion of patients immunised with nirsevimab among RSV bronchiolitis cases over the study period.**

**Figure S1. Respiratory syncytial virus (RSV) positivity rate for nasopharyngeal or salivary swabs collected in France during 2018-2019 to 2023-2024.**

**
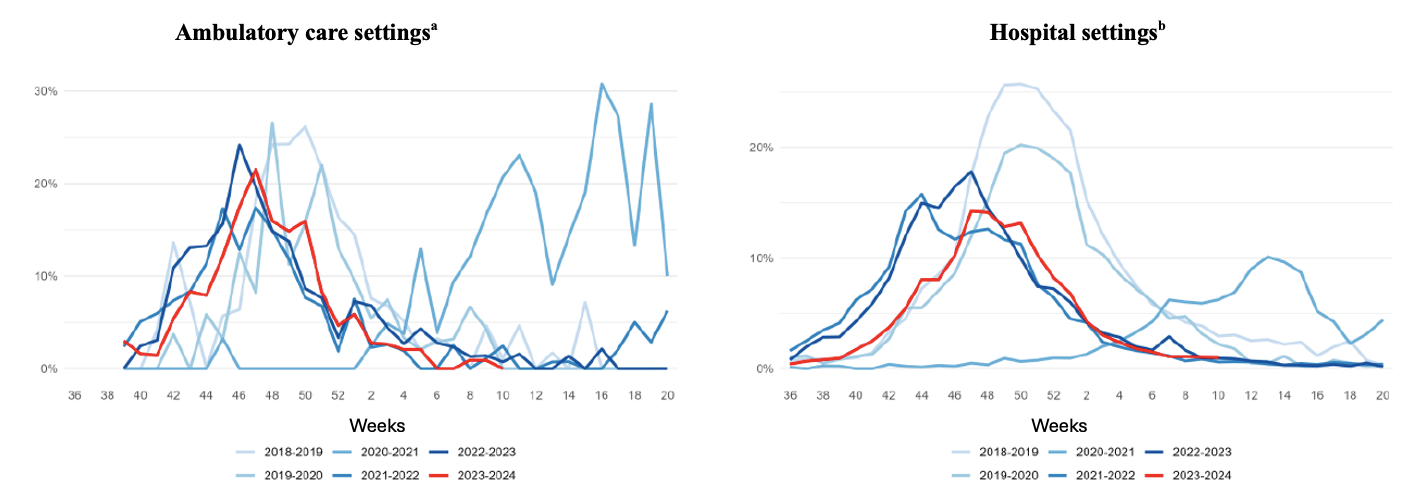
**

RSV positivity rate was calculated as the proportion (%) of positive samples over time (weeks), for all age groups (children and adults) and all symptoms combined.

^a^ Data obtained from the SENTINELLES network, a national research and health monitoring network for primary care involving general practitioners and private paediatricians throughout metropolitan France.

^b^ Data obtained from the RENAL network, a hospital laboratory-based national network.

Reference: Santé Publique France website. <https://www.santepubliquefrance.fr/maladies-et-traumatismes/maladies-et-infections-respiratoires/grippe/documents/bulletin-national/infections-respiratoires-aigues-grippe-bronchiolite-covid-19-.-bulletin-du-13-mars-2024>

Abbreviation: RSV, respiratory syncytial virus

**Supplementary information. The Paediatric and Ambulatory Research in Infectious diseases (PARI) network.**

The Paediatric and Ambulatory Research in Infectious diseases (PARI) network, established in 2017, is a surveillance system combining automated data extraction from primary care paediatricians trained in infectious diseases. It monitors various paediatric infections, for children under 16 years old. The network includes 107 paediatricians from the French Ambulatory Paediatric Association (AFPA) using AxiSanté 5, Infansoft software (CompuGroup Medical, France). These paediatricians receive specific training in infectious diseases and access to a dedicated e-learning website, automated disease frequency graphs, and weekly newsletters. The distribution of these paediatricians across the French territory are represented in the attached figure.

Participating paediatricians follow French guidelines and use point-of-care tests such as rapid diagnostic tests for various infections, based on clinical judgment. The study was approved by relevant French authorities and registered at ClinicalTrials.gov (NCT04471493). To date, 200 659 children have been included in this network.

Attached figure: Distribution of the paediatricians in the PARI network across the French territory.


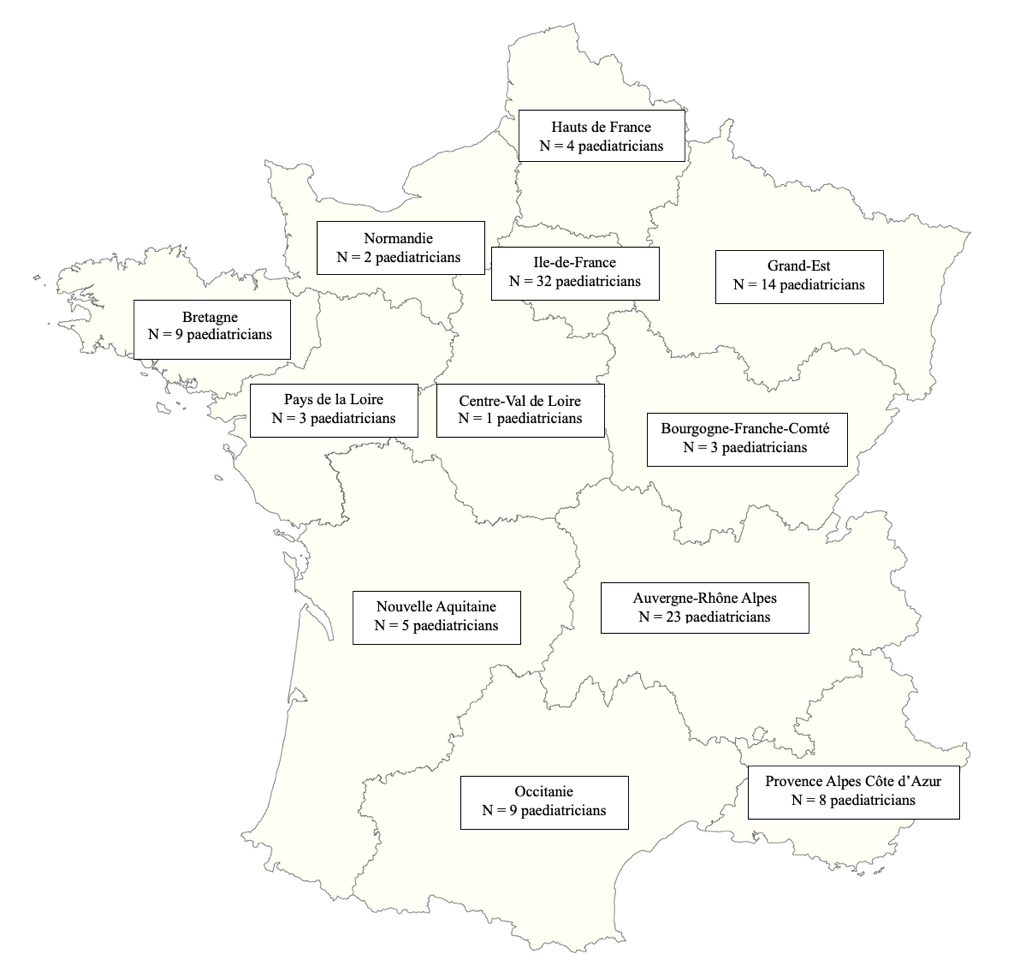


**References:**

- Cohen R, Bechet S, Gelbert N, Frandji B, Vie Le Sage F, Thiebault G et al: New Approach to the Surveillance of Pediatric Infectious Diseases From Ambulatory Pediatricians in the Digital Era. Pediatr Infect Dis J 2021, 40(7):674-680.
- Cohen R, Rybak A, Werner A, Bechet S, Desandes R, Hassid F et al: Trends in pediatric ambulatory community acquired infections before and during COVID-19 pandemic: A prospective multicentric surveillance study in France. Lancet Reg Health Eur 2022, 22:100497. A large-scale outbreak of hand, foot and mouth disease, France, as at 28 September 2021.
- Mirand A, Cohen R, Bisseux M, Tomba S, Sellem FC, Gelbert N, et al. Euro Surveill. 2021 Oct;26(43):2100978. doi: 10.2807/1560-7917.ES.2021.26.43.2100978. PMID: 34713796
- Cohen JF, Rybak A, Werner A, Kochert F, Cahn-Sellem F, Gelbert N et al. Surveillance of noninvasive group A Streptococcus infections in French ambulatory pediatrics before and during the COVID-19 pandemic: a prospective multicenter study from 2018-2022. Int J Infect Dis. 2023 Sep;134:135-141. doi: 10.1016/j.ijid.2023.06.003. Epub 2023 Jun 6. PMID: 37290573”
- Corinne Levy, Andreas Werner, Alexis Rybak, Stéphane Béchet, Christophe Batard, Frédéric Hassid, Roxane Desandes, Bruno Frandji, Naim Ouldali, Robert Cohen, Early impact of nirsevimab on ambulatory all-cause bronchiolitis: a prospective multicentric surveillance study in France, Journal of the Pediatric Infectious Diseases Society, 2024;, piae051, https://doi.org/10.1093/jpids/piae051

**Table S1. Sensitivity analyses for the effectiveness of nirsevimab against respiratory syncytial virus (RSV)-bronchiolitis.**

| **Sensitivity analysis** | **Regression Model** | **Explanatory variables** | **Comments** |
| --- | --- | --- | --- |
| Mixed effect model | Mixed effect model with multiple imputation. Investigator was treated as a random effect. | Nirsevimab immunisation status (dichotomous), birth weight (continuous), birth term (continuous), age in month (continuous), sex (dichotomous), history of bronchiolitis (discrete), number of children per household (discrete), childcare setting (discrete), and month of diagnosis (categorial). | All patients included. |
| Multivariate model excluding patients with history of bronchiolitis | Multivariate logistic regression model with multiple imputation. | Nirsevimab immunisation status (dichotomous), birth weight (continuous), birth term (continuous), age in month (continuous), sex (dichotomous), number of children per household (discrete), region (categorial), childcare setting (discrete), and month of diagnosis (categorial). | Patient with history of previous bronchiolitis excluded. |
| Complete case analysis | Multivariate logistic regression model. | Nirsevimab immunisation status (dichotomous), birth weight (continuous), birth term (continuous), age in month (continuous), sex (dichotomous), history of bronchiolitis (discrete), number of children per household (discrete), region (categorial), childcare setting (discrete), and month of diagnosis (categorial). | Patients with missing data for all covariates included in the model were excluded. |
| Propensity score analysis | Logistic regression model with multiple imputation. | Variables used to build the propensity score were patient birth weight (continuous), birth term (continuous), age in month (continuous), sex (dichotomous), history of bronchiolitis (discrete), number of children per household (discrete), region (categorial), childcare setting (discrete), and month of diagnosis (categorial). | Inverse-probability weighting (IPW) method: The probability – or propensity - of nirsevimab immunisation, given each patient’s characteristics, was calculated using a logistic regression model. Then, for each patient, the inverse of this probability was used as weights which were included in the regression model. See figure S2. |
| Transformation of continuous variables into categorial variables | Multivariate logistic regression model with multiple imputation. | Nirsevimab immunisation status (dichotomous), birth weight (categorial in quartile), birth term (dichotomised as birth < or ≥ 37 weeks of gestational age), age in month (continuous), sex (dichotomous), history of bronchiolitis (discrete), number of children per household (discrete), region (categorial), childcare setting (discrete), and month of diagnosis (categorial). | Multivariate model with birth term and birth weight as categorical variables instead of continuous variables. |
| Matched case-control analysis based on the diagnosis date with a 1:1 ratio | Multivariate logistic regression model with multiple imputation and matching case and control patients for the date of diagnosis | Nirsevimab immunisation status (dichotomous), birth weight (continuous), birth term (continuous), age in month (continuous), sex (dichotomous), history of bronchiolitis (discrete), number of children per household (discrete), region (categorial), childcare setting (discrete), and date of diagnosis (discrete). |  |
| Multivariate model adjusted for week of diagnosis | Multivariate logistic regression model with multiple imputation. | Nirsevimab immunisation status (dichotomous), birth weight (continuous), birth term (continuous), age in month (continuous), sex (dichotomous), history of bronchiolitis (discrete), number of children per household (discrete), region (categorial), childcare setting (discrete), and week of diagnosis (categorial). | Multivariate analysis adjusted for the week of diagnosis to better account for the rapidly increasing national coverage of nirsevimab over the study period. |
| Multivariate model adjusted for month of birth | Multivariate logistic regression model with multiple imputation. | Nirsevimab immunisation status (dichotomous), birth weight (continuous), birth term (continuous), month of birth (categorial), sex (dichotomous), history of bronchiolitis (discrete), number of children per household (discrete), region (categorial), childcare setting (discrete), and month of diagnosis (categorial). | Multivariate analysis adjusted for the month of birth to account for the higher likelihood of a severe RSV infection for infants born closer to the onset of the RSV season. |
| Multivariate model excluding patients with bronchiolitis within the 7 days of nirsevimab injection | Multivariate logistic regression model with multiple imputation. | Nirsevimab immunisation status (dichotomous), birth weight (continuous), birth term (continuous), age in month (continuous), sex (dichotomous), history of bronchiolitis (discrete), number of children per household (discrete), region (categorial), childcare setting (discrete), and month of diagnosis (categorial), delay between nirsevimab immunisation and diagnosis of bronchiolitis ≤ or > 7 days (dichotomised as 0 or 1). | Multivariate analysis to account for possible disease onset occurring before nirsevimab administration. |

**Figure S2. Covariate balance for the propensity score analysis.**


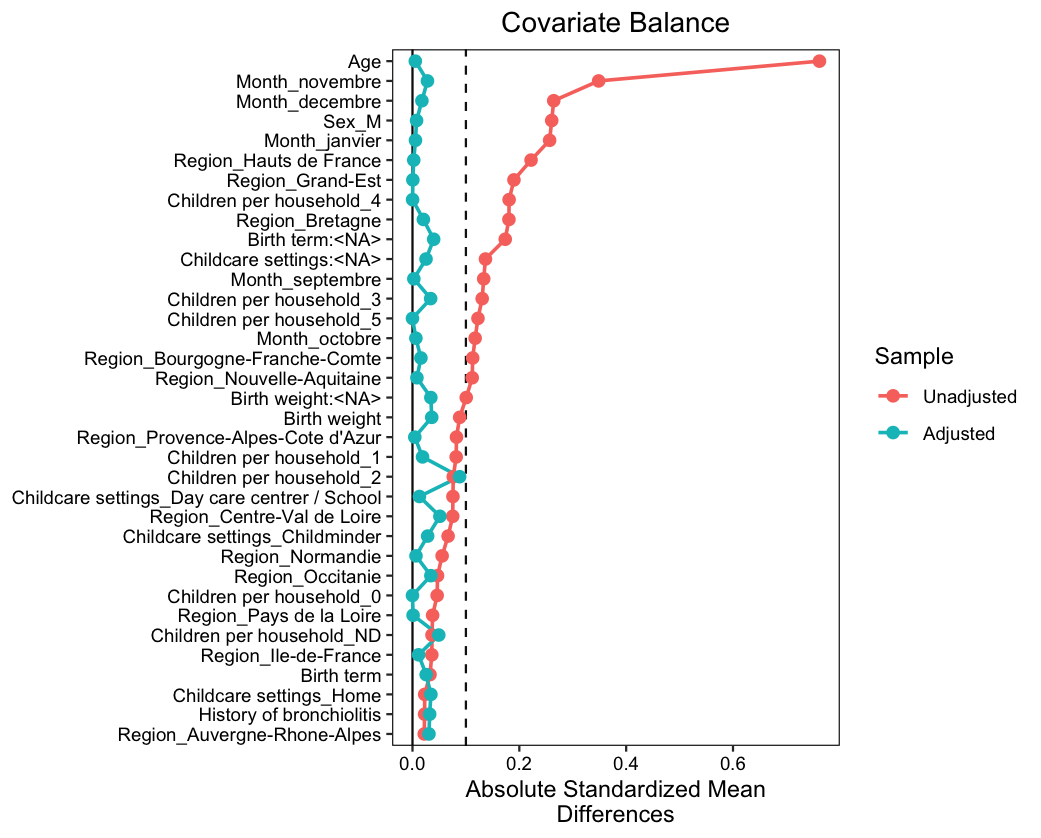


In this sensitivity analysis, a propensity score analysis using the inverse-probability of treatment weighting (IPTW) method was performed to better control for known confounders related to nirsevimab exposure.

Thus, the probability of receiving nirsevimab was estimated for each patient using a logistic regression model based on the following baseline covariates: 1) birth weight (continuous), 2) birth term (continuous), 3) age in months (continuous), 4) sex (dichotomous), 5) history of bronchiolitis (discrete), 6) number of children per household (discrete), 7) region (categorial), 8) childcare setting (discrete), and 9) month of diagnosis (categorial).

Then, to account for an initial covariate imbalance between treatment groups, we attributed a stabilised weight to each patient derived from the inverse probability of receiving nirsevimab using the previously estimated propensity scores. Finally, for each patient, the inverse of this probability was used as a weight to analyse the association between nirsevimab immunization status and case patient or control patient status with a quasi-binomial regression model. This figure presents the absolute standardised mean differences before and after IPTW for each covariate. All covariates included in the model presented standardised mean differences < 0.1, indicating that groups were well balanced after weighting.

Reference: Haukoos JS, Lewis RJ. The Propensity Score. JAMA. 2015 Oct 20;314(15):1637–8.)

**Table S2. Characteristics of included and excluded patients.**

|  | Included  N = 883 | Excluded  N= 915 | |
| --- | --- | --- | --- |
| Characteristic |  | **Nirsevimab status unknown**  **(N = 147)** | **Bronchiolitis without rapid antigen test**  **(N = 768)** |
| Age at diagnosis, median (IQR), months | 6.6 (4.6 -9.0) | 5.7 (4.1-6.9) | 7 (5.2-9.1) |
| Age group (%) |  |  |  |
| < 3 months | 88/883 (9.9) | 10/147 (6.8) | 39/768 (5.0) |
| ≥ 3 months | 795/883 (90.0) | 137/147 (93.2) | 729/768 (94.9) |
| Sex (%) |  |  |  |
| Male | 548/883 (62.0) | 90/147 (61.2) | 523/768 (68.1) |
| Female | 335/883 (37.9) | 57/147 (38.8) | 245/768 (31.9) |
| Birth term, median (IQR), weeks’ gestational age | 39 (38-40)  (NA = 173) | 39 (38-40)  (NA = 34) | 39 (38-40)  (NA = 203) |
| Preterm birth (%) | 65/710 (9.1) | 12/113 (10.6) | 48/565 (8.5) |
| Birth weight, median (IQR), kg | 3.245 (2.99-3.65)  (NA = 571) | 3.250 (3.01-3.58)  (NA = 91) | 3.274 (2.97-3.64)  (NA = 604) |
| History of bronchiolitis (%) |  |  |  |
| None | 777/883 (88.0) | 137/147 (93.2) | 652/768 (84.9) |
| 1 episode | 93/883 (10.5) | 10/147 (6.8) | 81/768 (10.5) |
| ≥ 2 episodes | 13/883 (1.4) | 0/147 | 35/768 (4.5) |
| Month of diagnosis (%) |  |  |  |
| September, 2023 | 45/883 (5.1) | 1/147 (0.7) | 54/768 (7) |
| October, 2023 | 172/883 (19.5) | 20/147 (13.6) | 145/768 (18.8) |
| November, 2023 | 340/883 (38.5) | 57/147 (38.7) | 207/768 (26.9) |
| December, 2023 | 243/883 (27.5) | 36/147 (24.5) | 223/768 (29.0) |
| January, 2024 | 83/883 (9.4) | 33/147 (22.4) | 139/768 (18.1) |
| Childcare setting (%) |  |  |  |
| Home | 284/631 (45.0) | 41/92 (44.5) | 226/550 (41.1) |
| Childminder | 123/631 (19.5) | 15/92 (16.3) | 232/550 (42.1) |
| Day care centre/School | 224/631 (35.5) | 36/92 (39.1) | 92/550 (16.7) |
| Children per household (%) |  |  |  |
| 1 | 211/595 (35.4) | 40/93 (43.0) | 219/529 (41.4) |
| 2 | 276/595 (46.3) | 39/93 (41.9) | 214/529 (40.4) |
| ≥ 3 | 108/595 (18.1) | 14/93 (15.0) | 96/529 (18.1) |
| RSV status |  |  |  |
| Yes | 453/883 (51.3) | 71/147 (48.3) | - |
| No | 430/883 (48.7) | 76/147 (51.7) | - |
| Nirsevimab immunisation status |  |  |  |
| Yes | 239/883 (27.0) | - | 134/768 (17.4) |
| No | 644/883 (72.9) | - | 634/768 (82.6) |

Abbreviations: IQR, interquartile range; RSV, respiratory syncytial virus

**Figure S3. Monthly number of case and control patients included over the study period.**


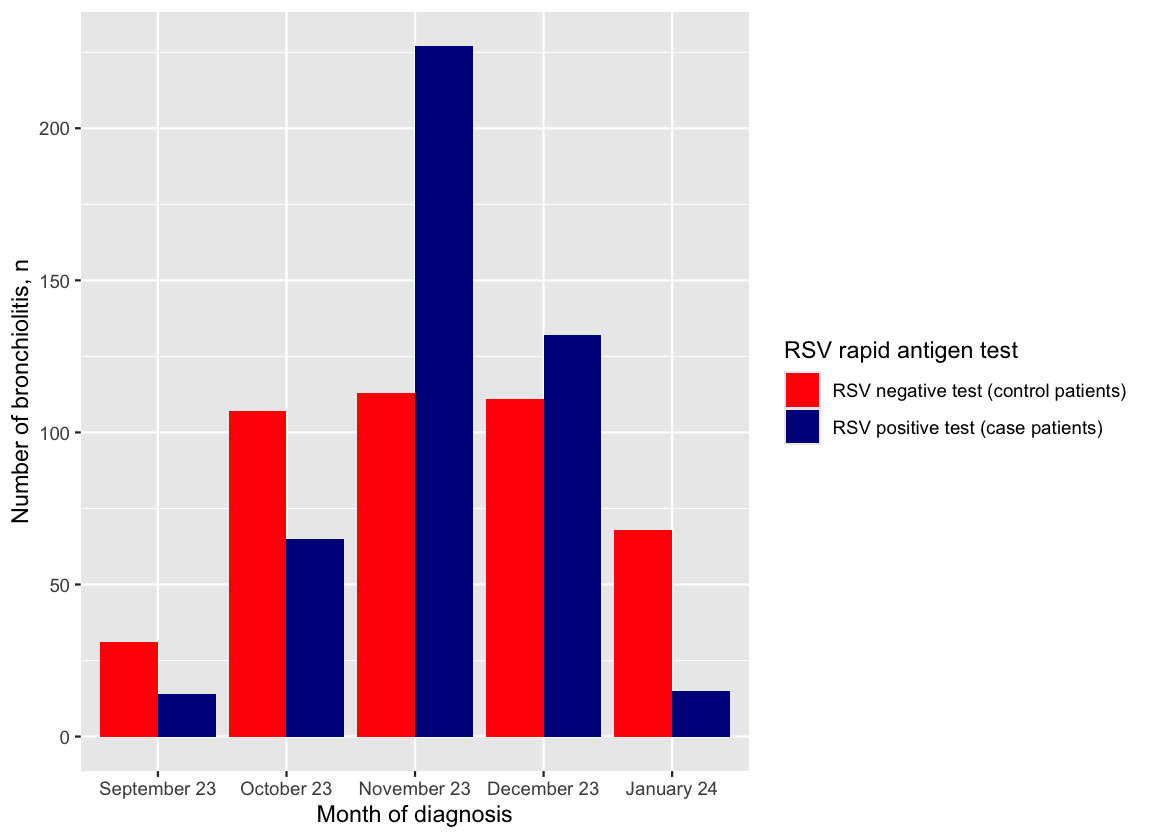


Abbreviation: RSV, respiratory syncytial virus

**Figure S4. Proportion of patients immunised with nirsevimab over the study period.**

**
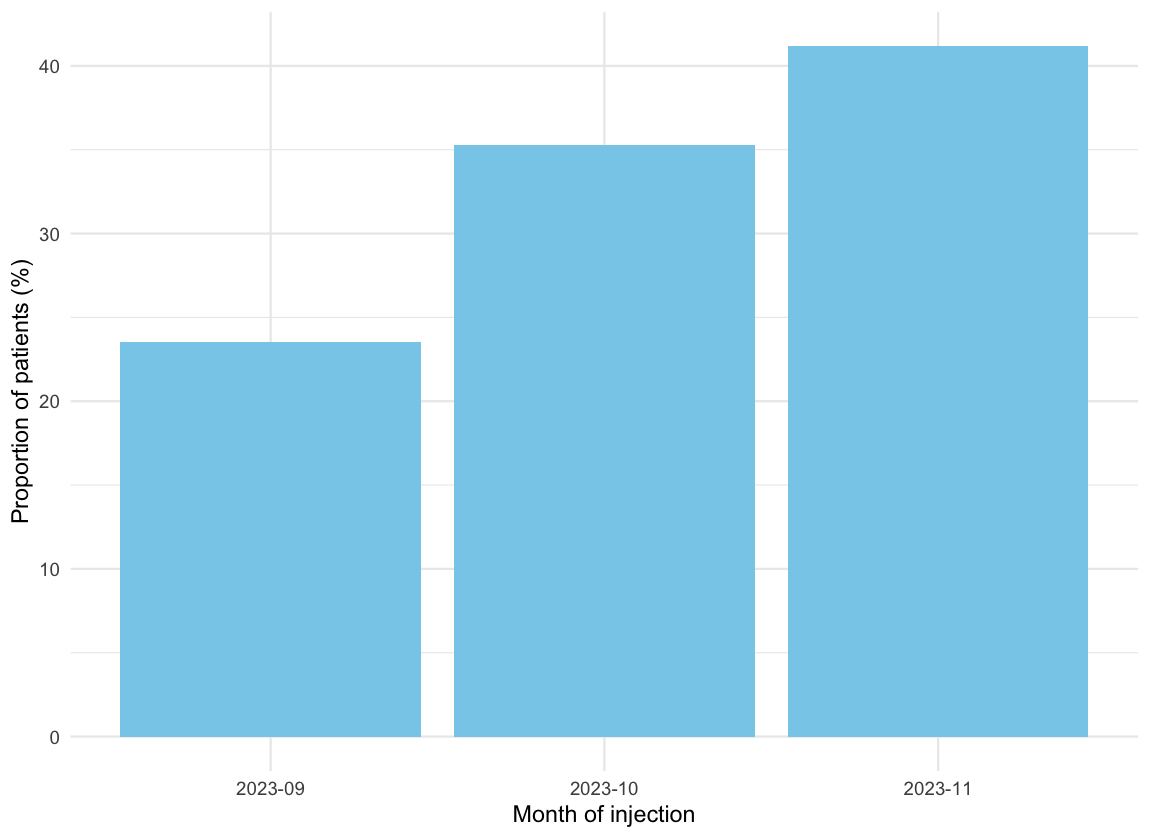
**

**Figure S5. Proportion of patients immunised with nirsevimab among RSV bronchiolitis cases over the study period.**


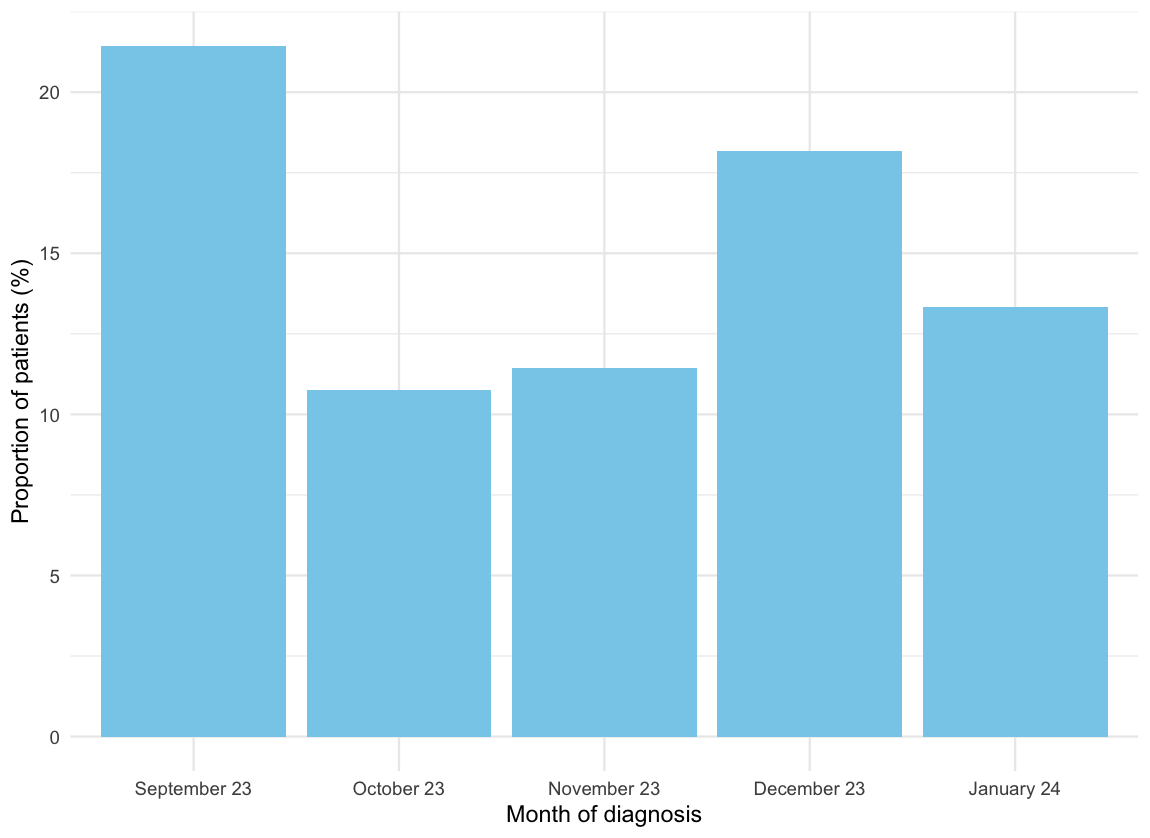

Supplement: Supplementary Tables and Figures [file mmc1.docx]
